# Lactate as a Cardiovascular Exerkine: Mechanisms, Signaling Pathways, and Clinical Implications

**DOI:** 10.3390/biom16070943

**Published:** 2026-06-24

**Authors:** Francesco Vari, Ilaria Serra, Elisa Bisconti, Daniele Vergara, Anna M. Giudetti

**Affiliations:** 1 Department of Biological and Environmental Sciences and Technologies (DiSTeBA), University of Salento, 73100 Lecce, Italy; francesco.vari@unisalento.it (F.V.); ilaria.serra@uniroma1.it (I.S.); elisa.bisconti@unisalento.it (E.B.); daniele.vergara@unisalento.it (D.V.); 2 Department of Physiology and Pharmacology “V. Erspamer”, Sapienza University of Rome, P.le Aldo Moro 5, 00185 Rome, Italy

**Keywords:** angiogenesis, atherosclerosis, cardiac function, cardiovascular system, exercise physiology, exerkine, HCAR1/GPR81, lactate, lactylation, liver, vascular tone

## Abstract

Lactate was traditionally considered a metabolic by-product of anaerobic glycolysis, mainly associated with tissue hypoxia and muscle fatigue. However, increasing evidence has redefined lactate as a multifunctional metabolic intermediate and signaling molecule involved in exercise-induced systemic adaptations. During physical activity, circulating lactate levels rise markedly when skeletal muscle production exceeds systemic clearance, allowing lactate to act as an exercise-responsive metabolite, or exerkine, and as a mediator of cardiometabolic adaptation. In the cardiovascular system, lactate serves not only as an efficient substrate for myocardial energy production but also as a regulator of vascular tone, endothelial function, angiogenesis, inflammation, and cardiac remodeling. These effects occur through receptor-dependent and receptor-independent mechanisms, including activation of hydroxycarboxylic acid receptor 1 (HCAR1/GPR81), modulation of intracellular redox balance, and histone or non-histone protein lactylation. This review summarizes current evidence on lactate in cardiovascular physiology and disease, focusing on myocardial lactate metabolism, HCAR1/GPR81 signaling, protein lactylation, extracellular vesicle communication, gut microbiota interactions, and therapeutic implications in heart failure, atherosclerosis, and diabetic cardiomyopathy. Although lactate is also produced under resting, postprandial, and pathological conditions, exercise is characterized by the amplitude and kinetics of lactatemia, coordinated hormonal and hemodynamic responses, and transient high-concentration signaling. These features support exercise-derived lactate as a context-dependent cardiovascular exerkine.

## 1. Introduction

Physical activity is one of the most effective non-pharmacological interventions for maintaining cardiovascular and metabolic health. Regular exercise reduces the incidence of cardiovascular diseases (CVD), metabolic disorders, and several forms of cancer, while improving cardiorespiratory fitness, cognitive function, and skeletal muscle performance [1]. At the metabolic level, lactate is now recognized as a central intermediate in interorgan substrate exchange and cellular signaling [2,3]. In recent years, growing attention has been focused on the molecular mediators responsible for these systemic benefits, particularly the class of exercise-induced signaling molecules collectively known as exerkines [4,5,6].

Among these, lactate has emerged as a central metabolic and signaling mediator. Historically considered a waste product generated during anaerobic glycolysis and associated with acidosis and muscle fatigue, lactate is now recognized as a key intermediate in interorgan metabolic communication. This paradigm shift was largely driven by the “cell-to-cell lactate shuttle” theory proposed by Brooks and colleagues, which demonstrated that lactate is continuously produced, exchanged, and oxidized among tissues under both physiological and pathological conditions [3].

During exercise, lactate production by working skeletal muscle rises substantially. Although the liver remains a major site of lactate clearance through gluconeogenesis, hepatic uptake becomes relatively insufficient during intense exercise, leading to a marked increase (about 10–25 mmol/L) in circulating lactate concentrations [7,8,9]. This systemic accumulation allows lactate to function as an exerkine capable of coordinating metabolic and cardiovascular adaptations.

The cardiovascular system is highly responsive to lactate. The heart efficiently oxidizes lactate as an energetic substrate, particularly during exercise, while blood vessels respond to lactate through changes in endothelial signaling, nitric oxide (NO) production, vascular tone, and angiogenesis [10,11,12,13]. Moreover, the discovery of the hydroxycarboxylic acid receptor 1 (HCAR1/GPR81) and the identification of protein lactylation as a novel post-translational modification have greatly expanded our understanding of lactate-mediated signaling in cardiovascular tissues [14,15].

Recent evidence further indicates that lactate influences cardiovascular biology through multiple interconnected mechanisms, including modulation of inflammation, immune cell polarization, extracellular vesicle (EV) signaling, and gut microbiota-derived metabolites. These findings position lactate at the intersection of metabolism, epigenetics, vascular biology, and exercise physiology.

This review provides an integrated overview of the role of lactate as a cardiovascular exerkine, with deliberate prioritization of the mechanistic pathways considered most central to exercise-induced cardiovascular adaptation. Specifically, we focus on three hierarchically organized tiers of lactate action: (i) its concentration-dependent receptor signaling via HCAR1/GPR81, which represents the most direct and pharmacologically tractable mechanism; (ii) its redox-epigenetic effects through protein lactylation, which translates metabolic state into durable gene-regulatory changes; and (iii) its systemic integrative role within the interorgan lactate shuttle, connecting skeletal muscle, liver, and cardiovascular tissues. We also examine the translational implications of targeting this lactate-cardiovascular axis in heart failure, atherosclerosis, and diabetic cardiomyopathy.

## 2. Lactate as a Constitutive Metabolic Signal and the Context-Dependent Nature of Exercise-Derived Lactatemia

Lactate is a ubiquitous metabolic intermediate produced constitutively across a wide range of physiological and pathological conditions, independent of any exercise stimulus. Under resting conditions, basal lactate production occurs continuously in red blood cells, skin, intestinal mucosa, renal medulla, and resting skeletal muscle, maintaining circulating concentrations at approximately 1–2 mmol/L, reflecting ongoing interorgan carbon exchange rather than metabolic stress [16]. At these concentrations, lactate already participates in constitutive signaling, including partial HCAR1 activation and tonic regulation of lipid metabolism and vascular homeostasis [16,17]. Carbohydrate ingestion raises lactate transiently through postprandial glycolytic flux, rarely exceeding 2–3 mmol/L and rapidly cleared by hepatic gluconeogenesis [16].

In hypoxic and ischemic conditions, lactate accumulation follows a fundamentally different mechanism, reflecting impaired mitochondrial oxidative capacity rather than enhanced metabolic flux, and is accompanied by acidosis and progressive tissue dysfunction, with levels exceeding 4–5 mmol/L in severe sepsis or shock carrying well-established prognostic significance [16]. Inflammatory states independently drive lactate production through the Warburg-like metabolic reprogramming of activated immune cells, where lactate functions as both a product and a regulator of the inflammatory response [16,18,19]; similarly, in the tumor microenvironment, constitutive aerobic glycolysis generates high local lactate concentrations that suppress anti-tumor immunity and promote angiogenesis [16]. Collectively, these observations establish that lactate concentration, dynamics, and biological consequences vary substantially depending on context, a distinction that becomes critical when defining the exerkine role of exercise-derived lactate.

Exercise produces a rapid, large-amplitude, and reversible increase in circulating lactate, from ~1–2 mmol/L at rest to 10–25 mmol/L during maximal effort, driven by enhanced glycolytic flux in contracting skeletal muscle exceeding systemic clearance capacity [20]. This magnitude and kinetics differ fundamentally from the modest or chronic elevations observed postprandially or in pathological conditions, where lactate reflects impaired oxidative metabolism or reduced clearance rather than a coordinated physiological response. Critically, exercise-induced lactatemia occurs within an integrated systemic milieu of increased energy demand, hemodynamic redistribution, autonomic activation, and coordinated co-release of other exerkines [4,5,7], a co-stimulatory environment not recruited when equivalent lactate concentrations arise from pathological sources. Furthermore, the high concentrations uniquely achieved during intense exercise access signaling regimes capable of engaging receptor-independent mechanisms such as widespread protein lactylation and redox reprogramming [14,16], which are not systematically activated at lower or dysregulated lactate levels. Therefore, this review defines lactate’s exerkine role in a context-dependent rather than molecule-specific sense: the transient, intensity-dependent lactatemia occurring within the physiologically intact, hormonally primed, and hemodynamically activated environment of exercise [4,16], capable of supporting adaptive cardiovascular remodeling through metabolic, receptor-mediated, redox-sensitive, immunomodulatory, angiogenic, and lactylation-dependent mechanisms [13,14,18,21], outcomes fundamentally distinct from those driven by pathological lactate accumulation. To organize these mechanisms, the following sections structure lactate-dependent cardiovascular adaptation around three regulatory levels: its role as a metabolic substrate for the myocardium [10,22,23]; its function as a signaling molecule through HCAR1/GPR81 activation, redox modulation, nitric oxide-dependent vascular responses, and inflammatory regulation [15,17,18]; and its contribution to longer-term remodeling via epigenetic and post-translational mechanisms, particularly histone and non-histone lactylation influencing endothelial activation, macrophage polarization, angiogenesis, and cardiac repair [14,24,25].

## 3. Exercise Modalities and Systemic Lactate Dynamics

### Aerobic Exercise, Resistance Training and High-Intensity Interval Training

Exercise modalities differ fundamentally in the lactate profiles they generate and in the cardiovascular adaptations they elicit. Aerobic exercise (AE), encompassing activities such as running, cycling, and swimming, depends predominantly on oxidative metabolism and produces a graded lactate response proportional to intensity: below the maximal lactate steady state (MLSS, defined as the highest exercise intensity at which blood lactate concentration remains stable over time), production and clearance remain balanced, whereas higher intensities drive rapid lactate accumulation through accelerated glycolytic flux [26]. Chronic AE induces well-characterized cardiovascular adaptations, including eccentric left ventricular hypertrophy, increased stroke volume, improved cardiorespiratory efficiency, and enhanced mitochondrial density and oxidative capacity of skeletal muscle [27,28].

Resistance training (RT) involves repeated muscular contractions against external loads and generates significant acute lactate spikes driven by the recruitment of type II glycolytic fibers during brief, forceful contractions against external loads; the magnitude of accumulation is highly protocol-dependent [29]. The cardiovascular consequences of this pattern differ substantially from those of AE. RT promotes concentric rather than eccentric left ventricular hypertrophy, reflecting pressure overload rather than volume overload as the dominant hemodynamic stimulus, and while chronic RT does reduce resting blood pressure, improve endothelial function [30], and enhance autonomic regulation, its effects on ventricular-arterial coupling and vascular adaptation remain less well characterized than those of aerobic modalities.

High-Intensity Interval Training (HIIT) consists of repeated bouts of short, high-intensity efforts, ranging from near-maximal to truly maximal, interspersed with recovery periods of varying duration and intensity. HIIT occupies a metabolically distinct position: it sequentially recruits phosphocreatine hydrolysis in the initial seconds of each interval and anaerobic glycolysis as interval duration extends, generating sustained and repeated lactatemia that simultaneously challenges aerobic and anaerobic systems. This structure allows oxygen uptake to approach maximal levels within each workout while promoting adaptations in muscular buffering capacity, metabolic flexibility, VO_2_max, and mitochondrial density, making it an efficient strategy for enhancing overall cardiorespiratory fitness [31,32].

Crucially, it remains unclear whether the acute lactate spikes characteristic of RT are sufficient to engage systemic signaling pathways to the same extent as the sustained lactatemia observed during HIIT or continuous AE. This question is particularly relevant given lactate’s emerging role as a myokine and exerkine mediating interorgan crosstalk, and it has direct implications for the comparative cardiovascular and metabolic prescription of these modalities [4,26,31,32]. Different exercise modalities generate distinct lactate kinetics and therefore may activate lactate-dependent cardiovascular signaling pathways to different extents.

## 4. The Liver as a Regulator of Systemic Lactate Availability

### Hepatic Lactate Metabolism: An Overview

During intense exercise, the muscles’ ATP demand outpaces oxidative phosphorylation capacity, driving a sharp rise in glycolysis. When oxygen availability is limited or mitochondrial uptake is impaired, pyruvate is redirected toward lactate production via lactate dehydrogenase (LDH), simultaneously regenerating NAD^+^ to sustain glycolytic flux. The resulting lactate is shuttled out of muscle fibers through monocarboxylate transporters (MCT): MCT4 exports it from fast-twitch fibers into the bloodstream, while MCT1 facilitates its uptake into metabolically active tissues such as the heart, slow-twitch muscle, and liver [3].

**Figure 1:**
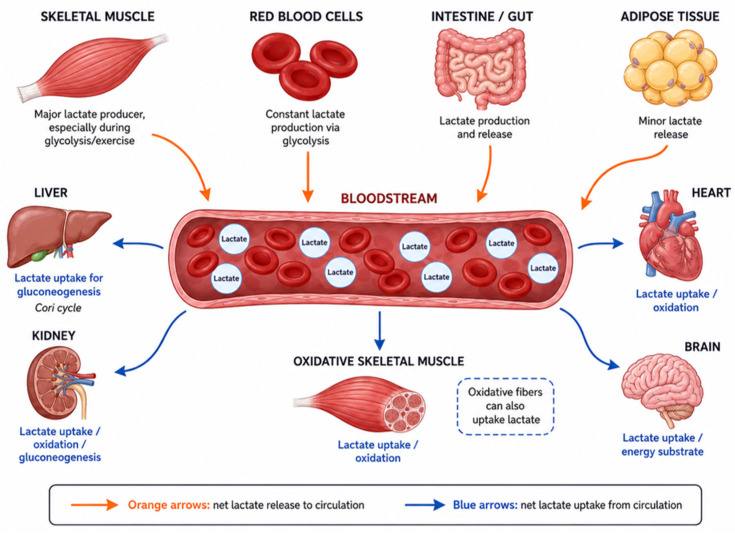
Schematic overview of circulating lactate distribution across tissues. Lactate enters the bloodstream from several sources, including skeletal muscle, red blood cells, intestine/gut, and adipose tissue, with skeletal muscle representing a major contributor during exercise. Circulating lactate is then taken up by metabolically active tissues, including the heart, liver, kidney, brain, and oxidative skeletal muscle, where it can be oxidized, used as an energy substrate, or converted into glucose through gluconeogenic pathways. Orange arrows indicate net lactate release into the circulation, whereas blue arrows indicate net lactate uptake from the circulation. Net lactate flux depends on physiological state, including rest, exercise, feeding, and hypoxia.

The resulting lactate is shuttled out of muscle fibers through monocarboxylate transporters (MCT): MCT4 exports it from fast-twitch fibers into the bloodstream, while MCT1 facilitates its uptake into metabolically active tissues such as the heart, slow-twitch muscle, and liver [33]. This circulating lactate pool reflects the dynamic balance between tissue-specific lactate release and uptake across organs (Figure 1).

**Figure 2:**
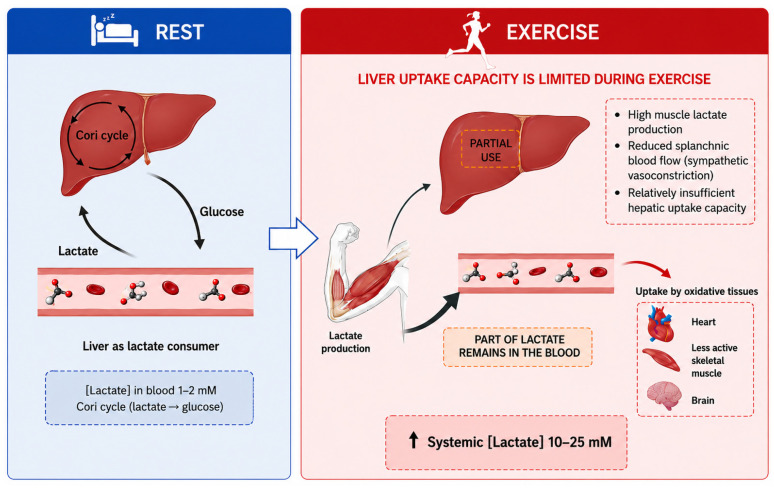
Distinct roles of the liver in lactate handling at rest and during exercise. At rest, the liver acts predominantly as a major lactate consumer, clearing circulating lactate from the blood and converting it into glucose, thereby maintaining low blood lactate concentrations (about 1–2 mM). During exercise, however, lactate production by working skeletal muscle rises markedly and can exceed the hepatic uptake capacity, thus remaining partially used by the liver. At the same time, reduced splanchnic blood flow further limits hepatic lactate clearance. As a result, only part of the circulating lactate is taken up by the liver, while a substantial fraction remains in the bloodstream and becomes available to other oxidative tissues, including the heart, skeletal muscle, and brain. This shift contributes to the increase in systemic lactate concentration observed during exercise (about 10–25 mM).

The liver is the organ with the greatest metabolic versatility in the human body. Under resting conditions, it acts as a major site of lactate clearance, extracting lactate from portal and systemic circulation and converting it to glucose via gluconeogenesis, a key component of the Cori cycle [7]. Hepatocytes express high levels of LDH isoforms, MCTs (primarily MCT1 and MCT2), and the full gluconeogenic enzymatic machinery, supporting an important role for the liver in systemic lactate removal at rest and during exercise (Figure 2) [20].

During exercise, this paradigm shifts substantially. As exercise intensity increases, lactate production by working skeletal muscles rises exponentially and exceeds the capacity of hepatic uptake and gluconeogenesis [20]. Although hepatic lactate uptake increases with exercise intensity, it remains quantitatively insufficient relative to peripheral release. Arteriovenous balance studies in humans demonstrate that hepatic lactate extraction accounts for only a small fraction of total lactate production, particularly during high-intensity exercise, where it may represent as little as ~10% of leg lactate output [34]. This relative limitation in hepatic clearance contributes to the progressive accumulation of lactate in the systemic circulation.

Excess lactate is redistributed and utilized by other tissues. According to the lactate shuttle concept, lactate serves as a key circulating metabolic intermediate, being rapidly taken up and oxidized by highly oxidative tissues such as the heart, as well as by less active skeletal muscle fibers (red fibers) [2,3]. The heart, in particular, has a high capacity for lactate oxidation and may rely on lactate as a major fuel during exercise. Thus, the rise in blood lactate during intense exercise primarily reflects a systemic imbalance between production and clearance.

In parallel, exercise-induced sympathetic vasoconstriction reduces splanchnic blood flow, thereby limiting hepatic perfusion and oxygen availability, which may impair hepatic lactate uptake and metabolism [35].

In conclusion, during intense exercise, the liver shifts from being the dominant lactate-clearing organ to one component of a broader systemic lactate redistribution network.

## 5. Lactate as an Exerkine: Conceptual Framework

### 5.1. Definition and Classification of Exerkines

The term “exerkine” was formally proposed to describe signaling moieties secreted in response to exercise that exert autocrine, paracrine, or endocrine effects on distant tissues [6]. Exerkines include a heterogeneous array of molecules: myokines (secreted by skeletal muscle), hepatokines (secreted by the liver), adipokines (secreted by adipose tissue), cardiokines (secreted by the heart), and small metabolites including lipids, amino acids, and organic acids. Well-characterized exerkines include irisin, interleukin-6 (IL-6), fibroblast growth factor 21 (FGF21), follistatin, and brain-derived neurotrophic factor (BDNF) [6].

**Figure 3:**
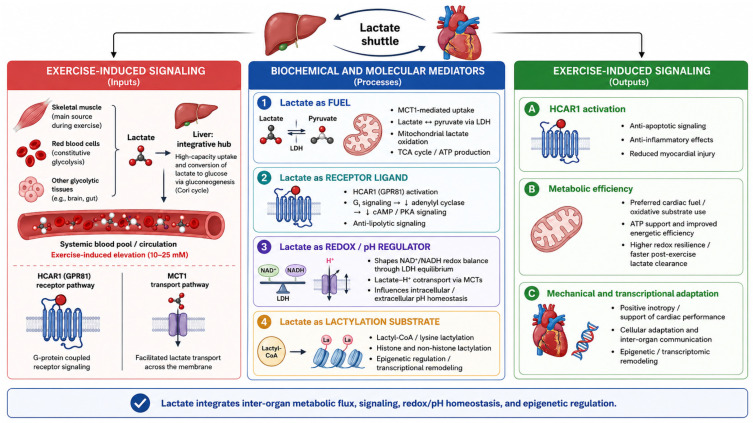
Diagram of lactate production, distribution, and biological actions during physical exercise. During exercise, lactate production increases mainly in contracting skeletal muscle, with additional contributions from red blood cells and other glycolytic tissues. When lactate production exceeds hepatic uptake and gluconeogenesis, circulating lactate levels rise markedly, particularly during high-intensity exercise. Within the lactate shuttle framework, lactate is redistributed through the systemic circulation to oxidative tissues, including the heart, where it acts both as an energetic substrate and as a signaling molecule. Lactate uptake through monocarboxylate (MCT) transporters, particularly MCT1, supports its conversion to pyruvate by lactate dehydrogenase and subsequent mitochondrial oxidation for ATP production. In parallel, lactate activates HCAR1/GPR81-dependent signaling, modulates redox and pH homeostasis, and contributes to lactylation-dependent regulation.

Lactate fulfills the key criteria defining an exerkine [4]. During exercise, its plasma concentration increases markedly, rising from ~1–2 mmol/L at rest to 10–25 mmol/L during maximal effort (Figure 3). Lactate, produced and released predominantly by working skeletal muscle and red blood cells, circulates systemically and is taken up by distant organs where it serves both metabolic and signaling functions within the framework of the lactate shuttle [2,3].

Crucially, lactate exerts its signaling action through specific receptors (HCAR1/GPR81) as well as non-receptor-mediated pathways, thereby modulating the function of diverse target organs [17,36]. A recent comprehensive review by Brooks and colleagues [37] has further proposed that lactate operates continuously as a signaling molecule not only during exercise but also at rest, following carbohydrate ingestion, during injury, and throughout pathological states, reinforcing its identity as a constitutive, concentration-dependent metabolic signal rather than merely an exercise-specific one.

### 5.2. Receptor and Non-Receptor Mechanisms of Lactate Signaling

The identification of HCAR1, a G-protein-coupled receptor (GPCR) activated by L-lactate, was a landmark in the field [15]. HCAR1 was initially characterized in adipocytes, where lactate-mediated HCAR1 activation suppresses lipolysis through inhibition of adenylyl cyclase and consequent reduction in intracellular cyclic AMP (cAMP) [36,37]. Subsequent studies revealed HCAR1 expression in a diverse range of tissues, including the heart, blood vessels, brain, and immune cells, suggesting a broader physiological role [38,39,40,41].

EC50 values for L-lactate acting on HCAR1 are in the range 1–5 mM [34], placing them within the physiological range of lactate concentrations achieved during moderate exercise [16]. This suggests that even submaximal exercise may generate sufficient circulating lactate to activate HCAR1 in selected target tissues, providing a mechanism by which the exercising body communicates metabolic state to the cardiovascular system in a graded, concentration-dependent fashion.

Importantly, lactate signaling appears to be concentration-dependent, with different ranges of circulating lactate likely engaging partially distinct cellular and molecular pathways across tissues. Under resting physiological conditions, blood lactate concentrations are generally maintained around ~1–2 mM, supporting basal interorgan lactate shuttling and constitutive metabolic communication [16,20]. At these concentrations, HCAR1 signaling is likely to be partial, context-dependent, and tissue-selective, potentially contributing to tonic regulation of lipid metabolism and vascular homeostasis [17,38].

During moderate or submaximal exercise, circulating lactate often rises into the ~3–5 mM range [16]. This range overlaps with the reported EC50 range for HCAR1 activation [15,17,41], suggesting that moderate exercise may provide a physiological context in which receptor-mediated lactate signaling becomes more relevant, in addition to lactate’s role as a metabolic substrate. In this range, lactate-sensitive pathways, including HCAR1-dependent mechanisms, may be engaged in receptor-expressing cells such as adipocytes, immune cells, vascular cells, and possibly cardiomyocytes, contributing to the regulation of lipid metabolism, inflammatory tone, vascular function, and angiogenic responses [18]. These concentrations are commonly observed during sustained endurance exercise below or near the individual lactate threshold, suggesting that moderate lactatemia may favor adaptive cardiovascular signaling without necessarily imposing severe metabolic stress [13,16,26].

By contrast, high-intensity exercise can elevate circulating lactate to substantially higher levels, in some cases reaching ~10–25 mM, particularly during repeated, sprint-like, or near-maximal efforts [31,32]. Under these conditions, the biochemical environment differs markedly, and receptor-independent mechanisms may become increasingly relevant. These may include changes in cellular redox balance and histone lactylation-related signaling [3,14]. Such mechanisms may contribute to epigenetic remodeling, metabolic adaptation, inflammatory modulation, and tissue-repair or angiogenic processes, although the direction and magnitude of these effects are likely to depend on duration, tissue context, oxygen availability, and mitochondrial oxidative capacity. Accordingly, while transient exercise-induced hyperlactatemia may support adaptive remodeling, persistent or excessive lactate accumulation, particularly in metabolically stressed or injured tissues, may contribute to maladaptive responses in a context-dependent manner [39].

Overall, this concentration-dependent framework supports a hierarchical rather than binary model of lactate signaling, in which distinct lactate ranges may preferentially engage metabolic, receptor-mediated, immunological, and epigenetic programs. This model may help explain why exercise modalities characterized by different lactate kinetics induce partially divergent cardiovascular adaptations [16,31,32].

Beyond its receptor-mediated and redox-dependent effects, lactate has recently been identified as a regulator of intracellular ion transport, adding a further dimension to its signaling repertoire. A landmark study by Daw and colleagues demonstrated that L-lactate acts as an intracellular second messenger triggering the rapid release of Mg^2+^ from endoplasmic reticulum (ER) stores, which in turn facilitates mitochondrial Mg^2+^ uptake via the inner mitochondrial membrane transporter Mrs2 [42]. This process is dose-dependent, temperature-sensitive, and mediated through intracellular rather than extracellular signals, establishing lactate as a direct ligand linking glycolytic flux to mitochondrial bioenergetics. Given that mMg^2+^ is an essential cofactor for TCA cycle enzymes, ATP synthase activity, and the formation of the biologically active Mg-ATP complex, lactate-driven ER-mitochondrial Mg^2+^ dynamics may represent a mechanism by which exercise-induced lactatemia directly augments mitochondrial oxidative capacity in cardiomyocytes. Acute treadmill exercise in mice has been shown to modulate cardiac gene expression in a lactate-dependent manner, including upregulation of PGC-1α, NRF-2, and COX-IV, effects that were attenuated by pharmacological blockade of MCTs [43]. Furthermore, lactate has been shown to act as a major upstream activator of AMPK and PGC-1α signaling during exercise, linking metabolic flux to mitochondrial biogenesis and adaptive remodeling [4].

### 5.3. Lactate as an Immunometabolic and Epigenetic Signal

An important immunological dimension of HCAR1 signaling has recently come into focus. Lactate binding to HCAR1 has been shown to downregulate Toll-like receptor-induced activation of the NLRP3 (NOD-, LRR- and pyrin domain-containing protein 3) inflammasome and reduce secretion of interleukin-1β (IL-1β), via an Arrestin-β2-dependent pathway [18]. This anti-inflammasome activity of lactate-HCAR1 signaling has been demonstrated both in the context of exercise and in inflammatory pathologies including sepsis and acute pancreatitis [44,45]. Beyond its immunosuppressive role, lactate actively contributes to pancreatic tissue remodeling by steering macrophage polarization toward a pro-repair phenotype. This effect is mechanistically linked to the promotion of lactylation and the concurrent inhibition of JAK2-STAT1 signaling, resulting in reduced inflammatory burden and enhanced tissue recovery, thus identifying a potentially exploitable target for the management of acute pancreatitis [46].

In the cardiovascular context, NLRP3 inflammasome activation is a driver of cardiac hypertrophy, atherosclerotic plaque instability, and myocardial injury following ischemia–reperfusion [47]. Exercise-derived lactate may thus contribute to cardiovascular protection partly through HCAR1-mediated NLRP3 suppression, adding an immunometabolic dimension to the lactate-cardiovascular exerkine axis. In addition to HCAR1-dependent signaling, lactate exerts GPR81-independent effects on macrophage metabolism, attenuating the pro-inflammatory response elicited by lipopolysaccharide (LPS) [19].

Beyond HCAR1, lactate exerts signaling effects through several receptor-independent mechanisms. As a weak acid (pKa ~3.86), lactate at physiological pH exists predominantly in its dissociated anionic form (lactate anion), which modulates intracellular and extracellular pH [48]. While historically attributed entirely to the accompanying protons, recent evidence indicates that lactate itself, independently of pH, can modulate ion channel activity, enzyme conformation, and redox state [49].

Lactate also serves as a substrate for post-translational protein modification: “lactylation” of lysine residues on histones and non-histone proteins, first described by Zhang and colleagues in 2019 [14], represents a newly recognized epigenetic mechanism through which lactate modifies gene expression in target cells, including cardiomyocytes and endothelial cells.

### 5.4. Interaction Between Lactate and Other Exerkines

Exercise induces the coordinated release of multiple exerkines that contribute to systemic adaptation [6]. Although lactate is increasingly recognized as a major exercise-responsive signaling metabolite [4,16], its interactions with other exerkines remain incompletely defined. Available evidence suggests that lactate may interact with, or converge on, several canonical exercise-induced mediators, including IL-6, FNDC5/irisin, BDNF, and FGF21, although the directionality and tissue specificity of these interactions remain to be fully established [5,6].

One relevant interaction involves IL-6, a myokine released by contracting skeletal muscle during exercise, with circulating responses influenced by exercise intensity, duration, and muscle glycogen availability [50]. Lactate production may contribute to IL-6 release during strenuous exercise, including through lactate-dependent protease activity, although this mechanism should be interpreted as context-dependent rather than universal [50]. In turn, IL-6 participates in substrate mobilization and metabolic homeostasis, including effects on hepatic glucose output and lipid metabolism [5]. The temporal relationship between these mediators may be relevant: lactate can rise rapidly during high-intensity exercise, whereas IL-6 generally accumulates progressively during prolonged or strenuous exercise, suggesting that lactate could contribute to selected downstream cytokine responses without acting as a sole trigger [50].

Lactate also appears to interface with FNDC5/irisin-BDNF signaling. Experimental evidence indicates that lactate can cross the blood–brain barrier and promote hippocampal BDNF expression through SIRT1-dependent mechanisms [51]. In parallel, endurance exercise has been shown to induce hippocampal BDNF through a PGC-1α/FNDC5 pathway [52]. Since FNDC5 can be cleaved to generate irisin, these observations support the possibility of an integrated lactate–FNDC5/irisin–BDNF axis linking skeletal muscle metabolism to neural adaptation. However, direct evidence establishing a single linear pathway in humans remains limited, and its cardiovascular relevance is likely indirect, potentially involving autonomic regulation, endothelial function, and inflammatory modulation [6].

Similarly, lactate may converge with FGF21 signaling rather than directly regulate it. FGF21 is an exercise-responsive hepatokine involved in metabolic adaptation, although the magnitude and direction of its response may vary according to exercise modality, duration, training status, and metabolic context [53]. Both lactate and FGF21 have been linked to energetic stress, AMPK/PGC-1α-related pathways, and oxidative metabolism [16,53]. Whether lactate directly regulates hepatic FGF21 secretion, or whether FGF21 modulates systemic lactate kinetics, remains unclear. Therefore, their interaction is best described as potential metabolic crosstalk within the exercise-responsive endocrine network.

Overall, these observations support the concept that lactate is unlikely to act in isolation, but rather as part of an integrated exerkine signaling network characterized by temporal sequencing, tissue-specific interactions, and pathway convergence. Defining the hierarchy and coordination among exercise-induced exerkines may help clarify the systemic biology of exercise adaptation and support the development of more targeted exercise-mimetic therapeutic strategies [4,5,6,16].

## 6. Effect of Lactate on Cardiovascular Structure

### 6.1. Histone Lactylation

The discovery that lactate can function as an acyl donor for post-translational protein modification, a process termed “lactylation”, has fundamentally expanded the molecular mechanisms through which lactate plays a role in cell signaling and gene expression [21,54]. Indeed, L-lactate reacts with protein lysine residues via an acyl-CoA intermediate (lactoyl-CoA), forming stable N-lactoyl-lysine bonds. Histones, particularly H3K18, H3K9, H4K8, and H4K12, are major targets of lactylation, and lactylated histones correlate with active gene transcription at their genomic loci [14].

The enzymatic regulation of lactylation follows a “writer-eraser-reader” framework analogous to other epigenetic modifications. The histone acetyltransferases p300 and CBP (CREB-binding protein) have been identified as major lactyltransferases (“writers”), while class I histone deacetylases (HDAC1-3) serve as delactylases (“erasers”), removing the lactyl group from substrate lysines [14]. More recently, the aminoacyl-tRNA synthetases AARS1 and AARS2 were identified as additional lactyltransferases that sense L-lactate and catalyze lactylation of cGAS and other targets, linking cellular lactate sensing directly to innate immune and epigenetic regulation [55]. This expanding network of lactylation enzymes provides multiple druggable nodes for modulating lactate-dependent epigenetic signaling in cardiovascular and other tissues.

Recent studies have further expanded the relevance of histone lactylation in cardiovascular pathology. In pressure overload-induced cardiac hypertrophy and heart failure, increased H3K18 lactylation has been shown to promote TGFB2 transcription and activate PI3K/AKT/mTOR signaling, thereby linking enhanced glycolytic metabolism to maladaptive cardiac remodeling [56].

In the vascular compartment, lactate-driven lactylation has also been implicated in endothelial dysfunction and atherosclerosis, where FOXO1 lactylation promotes nuclear translocation and inflammatory gene expression under atheroprone flow conditions [57]. Moreover, H4K16 lactylation in vascular smooth muscle cells has been reported to drive a PDK1-lactate positive feedback loop, promoting metabolic remodeling and phenotypic switching in aortic aneurysm and dissection [58]. These recent findings reinforce the concept that lactylation is not merely a marker of lactate accumulation but an active regulatory mechanism connecting metabolic reprogramming with cardiovascular remodeling and disease progression.

### 6.2. Non-Histone Lactylation

Beyond histone targets, the non-histone lactylome of the cardiovascular system has recently begun to be characterized at a systems level. In healthy mouse hearts, a comprehensive lactylation atlas identified 1674 lactylation sites across 477 cardiac proteins under basal physiological conditions, with myosin-6 and titin representing the proteins with the greatest number of lactylated residues [59]. Following myocardial infarction, global protein lactylation increased markedly, with site-specific changes reflecting the metabolic shift from oxidative phosphorylation to glycolysis in the ischemic myocardium [59]. This lactylome atlas reveals the breadth of lactate-dependent protein regulation in the heart and highlights the need for systematic lactylome profiling across exercise states, disease conditions, and training protocols to fully understand the scope of lactate’s epigenetic influence in the cardiovascular system.

Lactylation of α-myosin heavy chain (α-MHC) at lysine residue K1897, regulated by p300 as acyltransferase and SIRT1 as delactylase, is essential for its interaction with Titin and for maintaining normal sarcomeric structure and function. This modification is reduced in heart failure, and its loss worsens cardiac dysfunction. Conversely, boosting intracellular lactate levels (via sodium lactate administration or lactate transporter inhibition) restores α-MHC K1897 lactylation and alleviates heart failure, revealing a direct link between cardiac metabolism and sarcomeric integrity [24].

The significance of non-histone lactylation in the heart extends beyond the sarcomere. CBP-mediated lactylation of Snail1 has been implicated in the regulation of endothelial-to-mesenchymal transition (EndoMT) following myocardial infarction, a process that contributes to myocardial fibrosis and cardiac dysfunction post-injury [60].

PKM2, a glycolytic enzyme expressed in macrophages, undergoes lactylation at K62, which enhances its pyruvate kinase activity and shifts macrophage metabolism away from the Warburg effect, facilitating polarization towards a pro-resolving M2 phenotype [61]. Lactate-dependent non-histone lactylation may also regulate autophagy during intense exercise. In skeletal muscle, increased lactate production during heavy exercise has been linked to lactylation of PIK3C3/VPS34, a key component of the autophagy initiation machinery, enhancing its kinase activity and promoting autophagic flux [62]. This mechanism provides an additional link between exercise-induced glycolytic activation, proteostatic remodeling, and muscle adaptation to metabolic stress.

Taken together, these examples demonstrate that non-histone protein lactylation constitutes an extensive and functionally diverse regulatory network encompassing sarcomeric structural proteins, transcription factors, and metabolic enzymes, with direct relevance to cardiac repair, vascular inflammation, and immune modulation.

## 7. Functional Cardiovascular Effects of Lactate

### 7.1. Lactate as an Energetic Substrate

Lactate plays a central role in myocardial homeostasis, acting not only as an energy substrate but also as a signaling molecule. Although the healthy heart primarily relies on fatty acid oxidation, lactate becomes a major fuel source during exercise, when its contribution to cardiac energy production markedly increases. Beyond metabolism, lactate regulates mitochondrial biogenesis, influences cardiomyocyte maturation and regeneration, modulates electrophysiological activity, and contributes to protection against oxidative stress. In aging hearts, impaired lactate oxidation is associated with metabolic remodeling and reduced cardiac function. Moreover, lactate shuttling between cardiomyocytes and cardiac fibroblasts is essential for maintaining metabolic balance and structural integrity within the myocardium [22].

The human heart can extract lactate from blood against a concentration gradient, driven by the steep electrochemical potential across the cardiomyocyte sarcolemma and the high expression of MCT1 and MCT4 in cardiac tissue [63].

However, the precise intracellular pathway of lactate oxidation remains a subject of ongoing debate. The intracellular lactate shuttle (ILS) model [64], together with the proposed mitochondrial lactate oxidation complex (mLOC) [65], posits that lactate can be directly transported into mitochondria and oxidized in close association with mitochondrial enzymes, thereby tightly coupling lactate utilization to oxidative phosphorylation. This framework is supported by structural and functional data demonstrating the colocalization of lactate dehydrogenase with mitochondrial components, as well as by tracer studies showing substantial mitochondrial lactate oxidation [66].

In contrast, alternative models argue that lactate must first be converted to pyruvate in the cytosol, with only pyruvate entering the mitochondrial matrix via the mitochondrial pyruvate carriers MPC1/2. In this view, the malate-aspartate shuttle plays a central role in transferring the reduced equivalents (NADH) generated during lactate oxidation into the mitochondria, thereby linking cytosolic lactate metabolism to mitochondrial respiration indirectly, rather than through direct mitochondrial lactate uptake [67].

Despite these mechanistic differences, there is broad agreement that lactate represents a highly efficient and rapidly mobilizable fuel for the heart, particularly under conditions of increased workload such as intense exercise [23]. Furthermore, lactate can be rapidly converted to pyruvate and then to acetyl-CoA, thereby supplying carbon to the tricarboxylic acid (TCA) cycle independently of upstream glycolytic flux and supporting energy provision during exercise.

Indeed, lactate availability, concentration gradients, and redox state appear to be key determinants of its preferential utilization. Importantly, endurance training enhances the capacity for lactate uptake and oxidation, further reinforcing its central role in cardiac energetics [23]. Resolving the relative contribution of direct mitochondrial lactate oxidation versus cytosolic conversion pathways remains an important objective for future research, with implications for understanding metabolic flexibility in both physiological and pathological states.

### 7.2. Lactate-Dependent Vascular Signaling

Beyond its role as a metabolic fuel, lactate exerts direct effects on cardiac and vascular function through multiple mechanisms. At the systemic level, lactate participates in the regulation of cardiac output during exercise through its contribution to the activation of the muscle metaboreflex, a powerful cardiovascular reflex triggered by the accumulation of interstitial metabolites, including lactate, in underperfused skeletal muscle [68]. Initial studies also proposed that lactate may contribute to the ventilatory response to exercise by activating the olfactory receptor OLFR78 expressed in carotid body glomus cells [69], though subsequent work has challenged this interpretation, suggesting that OLFR78 mediates hypoxic chemosensing through mechanisms not strictly dependent on lactate itself.

The most immediate vascular consequence of lactate elevation during exercise is arteriolar vasodilation, facilitating increased blood flow to working tissues. First proposed by Gaskell over a century ago and subsequently supported by extensive experimental evidence, lactate-induced vasodilation operates through multiple, vascular bed-specific mechanisms [70,71,72]. In porcine coronary arteries, lactate activates Ca^2+^-activated K^+^ channels (KCa channels), a self-protective response that may help sustain coronary perfusion during ischemia [72].

Lactate induces concentration-dependent coronary vasodilation and reduces myocardial contractility in isolated perfused rat hearts, without affecting heart rate. The coronary vasodilatory effect is attenuated by NO synthase inhibition and upon endothelium removal indicating that lactate-mediated coronary vasodilation is primarily mediated by endothelial NO release [73]. In the retinal microvasculature a similar NO-dependent dilation occurs through neuronal NOS (nNOS) activation [74]. In skeletal muscle, Fontes et al. identified a distinct lactate-AMPK-NOS1 signaling axis, whereby muscle-derived lactate activates AMPK and phosphorylates NOS1 in skeletal muscle cells, exerting an anticontractile effect on adjacent femoral arteries, an effect abolished by MCT inhibition [75]. In the cerebral microvasculature, GPR81/HCAR1 has emerged as a key lactate sensor, mediating dose-dependent regulation of the hyperemic response, likely through modulation of the NAD^+^/NADH redox state and downstream NO production [76].

**Figure 4:**
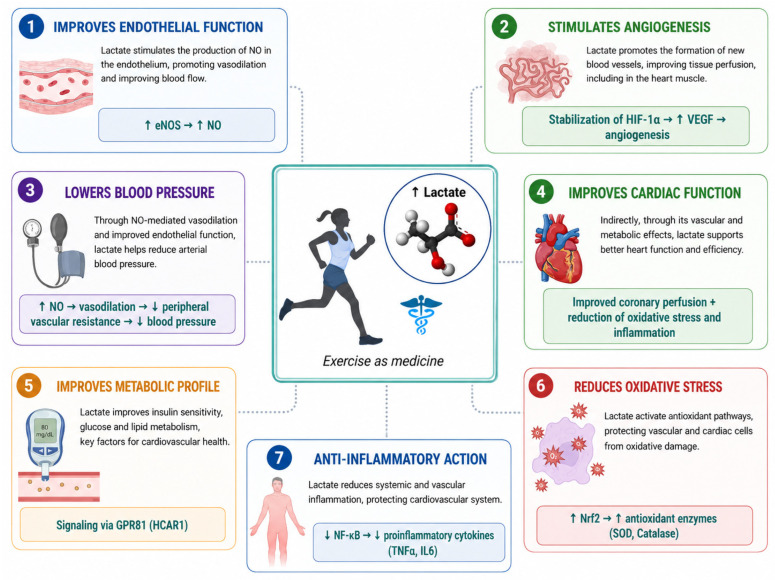
Schematic representation of the proposed cardioprotective actions of exercise-released lactate. Beyond its traditional role as a metabolic intermediate, lactate acts as a signaling molecule able to influence endothelial function, nitric oxide (NO)-dependent vascular tone, angiogenesis through HIF-1α/VEGF-related pathways, cardiac energetic balance, metabolic homeostasis via GPR81-mediated signaling, and inflammatory responses. The NO-related pathway indicates a putative mechanism by which lactate may support endothelial NO availability, favoring vasodilation, lowering peripheral vascular resistance, and potentially contributing to blood pressure regulation.

Beyond acute vasoregulation, lactate contributes to endothelial adaptation and vascular remodeling. Lactate promotes angiogenesis in endothelial cells through multiple mechanisms: it upregulates both VEGF and its receptor VEGFR2 through a poly-ADP ribosylation (PAR)-dependent pathway linked to reduced NAD^+^ levels [77], and it stabilizes HIF-1α while driving histone lactylation to further potentiate pro-angiogenic gene expression [78], collectively contributing to the vascular adaptations observed with exercise training. Exercise-induced lactylation has also been shown to suppress endothelial expression of pro-inflammatory adhesion molecules ICAM-1 and VCAM-1, and to reduce monocyte adhesion to the endothelium, through epigenetic reprogramming of endothelial gene expression [25]. Collectively, the cardioprotective actions of exercise-derived lactate are schematically summarized in Figure 4.

### 7.3. Immunometabolic and Microbiota-Mediated Effects

The gut microbiota has recently emerged as a regulator of cardiovascular health, acting through a complex interplay of microbial metabolites, immune modulation, and host metabolic pathways. Exercise can modulate gut microbial composition, and lactate may represent one molecular link within the gut-liver-cardiovascular axis [79,80].

Circulating lactate can influence the intestinal environment both directly and indirectly. Within the gut lumen, lactate can serve as a substrate for lactate-utilizing microbial taxa. In particular, *Veillonella* species have been shown to convert exercise-derived lactate into propionate, while broader microbiota-dependent lactate metabolism may contribute to short-chain fatty acid (SCFA) production axis [79,80]. These SCFAs exert well-established cardioprotective effects, including improvement of endothelial function, reduction in systemic inflammation, modulation of blood pressure via G-protein-coupled receptors such as GPR41 and GPR43, and enhancement of NO bioavailability [81,82,83,84].

Notably, exercise-induced elevations in circulating lactate have been associated with an enrichment of lactate-utilizing bacterial populations. In support of this concept, Scheiman et al. showed that *Veillonella atypica*, a gut bacterium enriched in marathon runners after racing, improves exercise performance by converting exercise-derived lactate, which crosses the gut epithelial barrier, into propionate. Inoculation of this strain in mice increased treadmill run time, an effect reproduced by intrarectal propionate alone, revealing a microbiome-encoded metabolic pathway that naturally enhances athletic performance [79].

Although still an emerging field, the interaction between lactate signaling and the gut microbiome represents a promising avenue for understanding the systemic adaptations to exercise and their cardiometabolic benefits.

## 8. Clinical Implications and Therapeutic Perspectives

### 8.1. Heart Failure and Metabolic Remodeling

CVD, encompassing coronary artery disease, heart failure (HF), hypertension, and peripheral arterial disease, remains the leading cause of global mortality [85]. Physical exercise is one of the most effective interventions for CVD prevention and rehabilitation and is consistently associated with lower cardiovascular risk and improved cardiometabolic profiles [86,87]. Understanding the molecular mechanisms mediating exercise’s cardiovascular benefits is a prerequisite for developing exercise-mimetic therapies that can extend these benefits to individuals unable to perform physical activity, including elderly, disabled, or severely ill patients.

Lactate supplementation strategies, including sodium lactate or oral lactate administration, have been explored mainly in preclinical and experimental settings for their potential to augment exercise-related metabolic adaptations [24,88].

HF is characterized by impaired cardiac energy metabolism, reduced exercise capacity, skeletal muscle wasting, and peripheral vascular dysfunction [89,90]. Alterations in lactate handling are well-documented in HF: at rest, patients with HF exhibit elevated circulating lactate levels, reflecting impaired hepatic and renal clearance and increased peripheral lactate production [91]. During exercise, the normal augmentation of lactate-driven cardiac fuel utilization is blunted in HF, as cardiomyocytes exhibit impaired mitochondrial oxidative capacity and reduced metabolic flexibility, with the pyruvate-lactate axis being critically disrupted [92].

### 8.2. Atherosclerosis and Endothelial Lactylation

Atherosclerosis, the leading pathological substrate of ischemic heart disease and stroke, is fundamentally an inflammatory disease of the arterial wall, driven by endothelial dysfunction, lipid accumulation, and macrophage foam cell formation. Physical exercise is robustly protective against atherosclerosis, and recent evidence identifies lactate-mediated protein lactylation as a key mechanistic bridge between exercise metabolism and anti-atherosclerotic vascular biology [93]. In an ApoE-deficient mouse model of atherosclerotic cardiovascular disease, exercise training was shown to promote Mecp2 lactylation at lysine 271 (Mecp2K271la) in aortic endothelial cells, concurrently downregulating pro-inflammatory mediators, including VCAM-1, ICAM-1, MCP-1, IL-1β, and IL-6, while upregulating eNOS expression. Mechanistic investigations through RNA sequencing and ChIP-qPCR revealed that Mecp2K271la directly occupies the chromatin of the Ereg gene promoter, repressing its transcription and thereby attenuating EGFR phosphorylation and downstream MAPK signaling. Importantly, exogenous lactate administration reproduced these effects in vivo, suppressing Ereg expression and MAPK activity and retarding atherosclerotic progression, reinforcing the concept that exercise-induced lactylation constitutes a post-translational mechanism linking physical activity to vascular protection [25]. Beyond the endothelial compartment, MeCP2 K271 lactylation also operates through engagement of the H3K36me3/RUNX1 axis in plaque macrophages, promoting their polarization toward a pro-repair M2 phenotype, stabilizing atherosclerotic lesions, and reducing cardiovascular risk. RUNX1 accordingly emerges as a promising pharmacological target for exercise-mimetic therapeutic strategies [94].

Additionally, the regulation of vascular smooth muscle cell phenotype by lactate may represent a further component of the lactate-atherosclerosis axis. In lactate-rich microenvironments, vascular smooth muscle cells have been reported to shift from a contractile to a synthetic phenotype, with increased migratory and proliferative properties [95].

While this transition may contribute to neointimal hyperplasia in pathological settings, it could also support adaptive vascular remodeling in response to exercise. The net effect is likely to depend on the magnitude and duration of lactate exposure, the vascular bed involved, and the local inflammatory milieu, underscoring the need for further mechanistic investigation.

### 8.3. Diabetic Cardiomyopathy

Regular exercise has been shown to upregulate genes related to oxidative phosphorylation in the diabetic heart, suggesting a beneficial modulation of molecular pathways involved in cardiac performance and metabolic function [96]. Diabetic cardiomyopathy (DCM) is a distinct cardiac disorder characterized predominantly by diastolic dysfunction, which may progress to systolic heart failure in advanced stages, even in the absence of overt coronary artery disease or hypertension. Emerging evidence suggests that altered lactate metabolism may contribute to DCM pathophysiology and may represent a potential therapeutic target. In this context, Ma and colleagues [97] reported elevated MCT4 in diabetic cardiomyocytes which boosts lactate efflux, disrupting lactate-pyruvate balance and fueling oxidative stress, inflammation, and heart damage. This also heightens macrophage infiltration via H4K12 lactylation. MCT4 inhibition in mice improved cardiac function; blood lactate can thus predict diastolic dysfunction, highlighting MCT4 as a therapeutic target. The interaction between lactate and insulin-regulated substrate metabolism is also relevant to diabetes-related remodeling. Recent in vivo evidence indicates that lactate homeostasis is maintained through coordinated regulation of glycolysis and lipolysis, processes that are strongly influenced by insulin action and determine the balance between lactate production, oxidation, and clearance [98]. In insulin-resistant or diabetic states, disruption of this balance may alter lactate turnover, impair metabolic flexibility, and contribute to cardiometabolic dysfunction.

The impact of exercise on this DCM-associated lactate dysregulation is an active area of investigation. Exercise training can produce controlled, transient elevations in circulating lactate and has been linked to AMPK/PGC-1α/GLUT4-related metabolic remodeling, improved insulin sensitivity, and mitochondrial adaptation in metabolic disease contexts [99]. In addition, lactate-driven lactylation may further resolve inflammation through GPR81-mediated M2 macrophage polarization, addressing both the metabolic and inflammatory dimensions of DCM. The interaction between exercise-derived lactate, MCT expression patterns, and macrophage polarization in the diabetic heart thus represents a mechanistically rich area with substantial therapeutic potential [97,99,100,101].

### 8.4. Therapeutic Perspectives

The recognition of lactate as a bioactive signaling molecule has opened new therapeutic perspectives in cardiovascular medicine (Table 1). Beyond its traditional role as a metabolic intermediate, lactate is increasingly viewed as a potential target for pharmacological and exercise-mimetic interventions aimed at reproducing some of the cardioprotective effects of physical activity in individuals unable to exercise adequately.

One possible approach involves targeting the lactate receptor HCAR1/GPR81. Activation of HCAR1 has been associated with anti-inflammatory, vasomodulatory, and metabolic effects, including suppression of NLRP3 inflammasome activation, modulation of endothelial signaling, and regulation of lipid metabolism [17,18]. Pharmacological HCAR1 agonists may therefore represent a novel strategy for reducing vascular inflammation, improving endothelial function, and attenuating adverse cardiac remodeling in conditions such as heart failure and atherosclerosis. However, the tissue-specific consequences of chronic HCAR1 activation remain incompletely understood, and further studies are needed to clarify receptor desensitization, downstream signaling specificity, and long-term safety.

Another emerging strategy is lactate supplementation. Administration of sodium lactate or lactate-enriched formulations has shown beneficial metabolic and cardioprotective effects in preclinical studies, including improved cardiac energetics, enhanced angiogenic signaling, and restoration of protective protein lactylation patterns [24,88]. Because lactate is readily utilized by the myocardium as an oxidative substrate, exogenous lactate supplementation could potentially support cardiac metabolism under conditions characterized by impaired metabolic flexibility, such as heart failure or ischemic injury. Nevertheless, the optimal dosing, route of administration, and safety profile of chronic lactate supplementation in humans remain to be established.

Modulation of MCTs, particularly MCT1 and MCT4, also represents a promising therapeutic avenue. These transporters regulate lactate exchange between cells and tissues and are essential for intracellular lactate shuttling and mitochondrial oxidation [63].

Altered MCT expression has been implicated in heart failure, diabetic cardiomyopathy, and vascular dysfunction [97]. Strategies aimed at restoring physiological lactate transport may improve myocardial energetic efficiency, reduce intracellular metabolic stress, and normalize lactate-dependent signaling pathways. In experimental models, inhibition of pathological MCT4 overexpression improved cardiac dysfunction and reduced inflammation in diabetic cardiomyopathy [97], highlighting the therapeutic potential of targeting lactate transport dynamics.

The concept of “exercise-mimetic” therapies has gained increasing attention in recent years. Since many individuals with advanced cardiovascular disease, frailty, or disability cannot perform sufficient physical activity, pharmacological interventions capable of reproducing exercise-induced molecular adaptations are highly attractive. Lactate signaling pathways represent a particularly compelling target in this context because they integrate metabolic, vascular, inflammatory, and epigenetic responses to exercise. Therapeutic strategies combining controlled lactate elevation, HCAR1 activation, AMPK signaling modulation, and mitochondrial metabolic reprogramming may eventually reproduce selected benefits of exercise training without requiring high physical workload.

Finally, growing interest surrounds the possibility of targeting protein lactylation as an epigenetic therapeutic strategy. Histone and non-histone lactylation regulate multiple cardiovascular processes, including endothelial inflammation, macrophage polarization, sarcomeric integrity, and post-ischemic remodeling [24,25,60]. Pharmacological modulation of lactylation “writers” and “erasers,” including p300/CBP, HDACs, SIRT1, and the recently identified AARS1/2 lactyltransferases, may offer novel approaches for controlling maladaptive cardiovascular remodeling [55]. Although this field remains at an early stage, selective modulation of lactylation pathways could eventually allow precise manipulation of exercise-responsive gene programs in cardiovascular tissues.

Overall, these emerging therapeutic strategies support the concept that the lactate signaling network may represent a translational bridge between exercise physiology and cardiovascular medicine. Further mechanistic studies and clinical trials will be necessary to determine whether modulation of lactate metabolism and signaling can be safely and effectively harnessed for cardiovascular prevention and therapy.

## 9. Future Directions and Open Questions

Despite substantial progress, many critical questions regarding lactate as a cardiovascular exerkine remain unanswered. First, the precise amount of systemic lactate during different exercise modalities and intensities in humans has not been rigorously defined. The technical challenges of hepatic vein catheterization during maximal exercise have limited human data, and isotopic tracer studies capable of distinguishing hepatic from muscular lactate sources are needed [20,34,35].

Second, the temporal and dose–response characteristics of HCAR1 signaling in human cardiovascular tissues remain poorly characterized [15,17,38]. Key questions include: what is the threshold lactate concentration required for meaningful HCAR1 activation in the intact coronary circulation? How does repeated HCAR1 activation with exercise training alter receptor expression and downstream signaling in cardiomyocytes and endothelial cells? Does HCAR1 desensitization occur with chronic lactate elevation, and if so, does this limit the cardiovascular benefits of high-lactate exercise protocols?

Third, the lactylome of the exercised human heart, the complete inventory of proteins modified by lactylation in response to exercise, has not been systematically characterized in vivo [14,59]. Proteome-wide lactylation profiling using mass spectrometry, combined with gene expression analyses and functional cardiac imaging, will be essential to delineate the full scope of epigenetic cardiovascular reprogramming by exercise-induced lactate. The identification of the enzymes responsible for site-specific cardiac protein lactylation, the writer-eraser-reader network, and their modulation by exercise intensity, duration, and training status is a particularly pressing research priority, given the potential of targeting these enzymes therapeutically [21,54,55].

Fourth, the interaction between lactate and other exercise-induced hepatokines, including FGF21, fetuin A, and SHBG, and their combined effects on cardiovascular physiology warrant investigation [6,53]. The liver represents a multifunctional endocrine organ during exercise, and understanding the integrated hepatokine response to exercise will be necessary to fully exploit the liver-cardiovascular axis for therapeutic benefit.

Fifth, the role of sex and aging in modulating lactate production, clearance, and cardiovascular lactate responsiveness deserves dedicated investigation [1,20]. There is indirect evidence suggesting that age-related changes in mitochondrial function and lactate transport may impair the cardiomyocyte’s ability to extract and utilize circulating lactate efficiently [22,63]. Exercise-induced lactylation responses may also be altered in older individuals, potentially limiting cardiovascular adaptations to training, although direct evidence remains limited [21]. Understanding how aging alters the lactate exerkine axis could inform exercise prescription strategies tailored to older populations at highest cardiovascular risk.

Sixth, the anti-atherosclerotic effects of lactate-driven protein lactylation, particularly Mecp2 K271la, observed in animal models require validation in human clinical studies [25,94]. Correlations between exercise-induced lactate peaks, circulating markers of Mecp2 lactylation, and indices of atherosclerotic burden, such as carotid intima-media thickness or coronary calcium scores, would provide clinically translatable evidence for the lactate-atherosclerosis axis in humans. Furthermore, whether exogenous lactate or HCAR1 agonist administration can replicate the anti-atherosclerotic effects of exercise in patients with established atherosclerotic disease is a key unanswered clinical question [25].

## 10. Conclusions

The concept of lactate as a simple by-product of anaerobic metabolism has been replaced by a more integrated view in which lactate acts as a metabolic substrate, signaling molecule, and epigenetic regulator. Exercise-induced increases in circulating lactate may contribute to cardiovascular adaptation through coordinated effects on myocardial energetics, vascular function, inflammation, angiogenesis, and gene regulation.

The identification of HCAR1/GPR81 signaling, mitochondrial lactate oxidation pathways, and histone and non-histone protein lactylation have provided mechanistic insight into how exercise-derived lactate influences cardiovascular physiology. These discoveries also suggest that lactate-mediated signaling may represent a promising therapeutic target in conditions characterized by metabolic and vascular dysfunction, including heart failure, atherosclerosis, and diabetic cardiomyopathy.

Importantly, lactate should no longer be viewed exclusively as a marker of metabolic stress, but rather as a central mediator of interorgan communication during exercise. The integration of lactate signaling with immune regulation and gut microbiota-derived pathways further highlights its systemic role in cardiometabolic homeostasis.

## Figures and Tables

**Table 1:** Candidate therapeutic strategies targeting lactate metabolism and signaling in cardiovascular medicine.

Therapeutic Strategy	Main Target/Mechanism	Proposed Cardiovascular Effects	Current Evidence	Main Limitations
HCAR1/GPR81 agonists	Activation of lactate receptor signaling	Anti-inflammatory effects, suppression of NLRP3 inflammasome, endothelial protection, modulation of vascular tone	Mainly pre-clinical studies [27,31,35]	Tissue-specific effects incompletely understood; possible receptor desensitization
Exogenous lactate supplementation (e.g., sodium lactate)	Increased systemic lactate availability and metabolic signaling	Improved cardiac energetics, angiogenesis, restoration of protective lactylation patterns	Preclinical studies and early translational exploration [51,87]	Optimal dose, route, and long-term safety remain unclear
MCT modulation (MCT1/MCT4)	Regulation of lactate transport and intracellular shuttling	Improved myocardial metabolic flexibility, reduced oxidative stress and inflammation	Experimental animal models [60,96]	Risk of disrupting physiological lactate exchange; tissue specificity required
Lactylation modulators	Targeting lactylation writers/erasers (p300/CBP, HDACs, SIRT1, AARS1/2)	Regulation of endothelial inflammation, macrophage polarization, sarcomeric integrity, post-ischemic remodeling	Early mechanistic/preclinical evidence [49,51,52,77]	Field still immature; limited pharmacological specificity
Exercise-mimetic metabolic therapies	Combined modulation of lactate-HCAR1-AMPK pathways	Partial reproduction of exercise-induced cardiovascular adaptations	Conceptual and early preclinical evidence [4,5,6,29]	Difficulty reproducing the multisystem complexity of exercise
Microbiota-directed lactate strategies	Enhancement of lactate-utilizing bacterial pathways	Increased SCFA production, reduced inflammation, improved vascular function	Emerging preclinical evidence [78,79,80,81,82,83]	High interindividual microbiota variability

## Data Availability

No new data were created or analyzed in this study. Data sharing is not applicable to this article.

## Abbreviations

The following abbreviations are used in this manuscript:

α-MHC	Alpha-myosin heavy chain
AARS1/AARS2	Aminoacyl-tRNA synthetases 1/2
AMPK	AMP-activated protein kinase
ATP	Adenosine triphosphate
BDNF	Brain-derived neurotrophic factor
cAMP	Cyclic adenosine monophosphate
CBP	CREB-binding protein
CVD	Cardiovascular disease
DCM	Diabetic cardiomyopathy
eNOS	Endothelial nitric oxide synthase
EndoMT	Endothelial-to-mesenchymal transition
EGFR	Epidermal growth factor receptor
ERK1/2	Extracellular signal-regulated kinase 1/2
EVs	Extracellular vesicles
FGF21	Fibroblast growth factor 21
GLUT4	Glucose transporter type 4
GPR	G protein-coupled receptor
GPCR	G protein-coupled receptor
HCAR1	Hydroxycarboxylic acid receptor 1
HDAC1-3	Histone deacetylases 1–3
HF	Heart failure
HIF-1α	Hypoxia-inducible factor 1-alpha
HIIT	High-intensity interval training
IL-1β	Interleukin-1 beta
IL-6	Interleukin-6
ILS	Intracellular lactate shuttle
LDH	Lactate dehydrogenase
LT	Lactate threshold
MAPK	Mitogen-activated protein kinase
MCT	Monocarboxylate transporter (MCT1, MCT2, MCT4)
Mecp2	Methyl-CpG-binding protein 2
mLDH	Mitochondrial lactate dehydrogenase
mLOC	Mitochondrial lactate oxidation complex
mMCT1	Mitochondrial monocarboxylate transporter 1
MPC1/MPC2	Mitochondrial pyruvate carriers 1/2
NAD^+^	Nicotinamide adenine dinucleotide (oxidized form)
NADH	Nicotinamide adenine dinucleotide (reduced form)
NLRP3	NOD-, LRR- and pyrin domain-containing protein 3
NO	Nitric oxide
OLFR	Olfactory receptor
PGC-1α	Peroxisome proliferator-activated receptor gamma coactivator 1-alpha
PI3K	Phosphoinositide 3-kinase
PKM2	Pyruvate Kinase M2
ROS	Reactive oxygen species
RUNX1	RUNX family transcription factor 1
SCFAs	Short-chain fatty acids
SIRT	Sirtuin
T2DM	Type 2 diabetes mellitus
TCA	Tricarboxylic acid (cycle)
VEGF	Vascular endothelial growth factor
VEGFR2	VEGF receptor 2
VO_2_max	Maximum oxygen consumption
